# Efficacy and Safety of Acupuncture at Tianshu (ST25) for Functional Constipation: Evidence from 10 Randomized Controlled Trials

**DOI:** 10.1155/2020/2171587

**Published:** 2020-11-06

**Authors:** Pengfan Li, Yue Luo, Qi Wang, Shi Shu, Kanjun Chen, Donghai Yu, Chunxiang Fan

**Affiliations:** ^1^Department of Traditional Chinese Medicine, Punan Hospital, Pudong New District, Shanghai 200125, China; ^2^Department of Dermatology, Yueyang Hospital of Integrated Traditional Chinese and Western Medicine, Shanghai University of Traditional Chinese Medicine, Shanghai 200437, China; ^3^Department of Traditional Chinese Medicine, Shuguang Hospital Affiliated to Shanghai University of Traditional Chinese Medicine, Shanghai 201203, China; ^4^Department of Traditional Chinese Medicine, Shanghai Seventh People's Hospital, Shanghai University of Traditional Chinese Medicine, Shanghai 200137, China

## Abstract

**Objective:**

To evaluate the evidence for the efficacy and safety of acupuncture at Tianshu (ST25) for functional constipation (FC).

**Methods:**

We systematically searched seven databases to identify randomized controlled trials of acupuncture at ST25 alone or in combination with conventional therapy in the treatment of FC. Risk ratios (RRs) and mean differences (MDs) were calculated using RevMan 5.3 with 95% confidence interval (CI).

**Results:**

The study included ten trials with 1568 participants. Meta-analysis showed that the Cleveland Constipation Score (CCS) for deep needling was significantly lower than that for lactulose (deep needling with low-frequency dilatational wave: MD −0.58, 95% CI −0.94 to −0.22; deep needling with sparse wave: MD −3.67, 95% CI −6.40 to −0.94; deep needling with high-frequency dilatational wave: MD −3.42, 95% CI −5.03 to −1.81). Furthermore, CCS for shallow needling with high-frequency dilatational wave was lower than that for lactulose (MD −1.77, 95% CI −3.40 to −0.14). In addition, when deep needling was combined with high-frequency dilatational wave, the weekly frequency of spontaneous defecation (FSD) was significantly higher than that for lactulose (MD 1.57, 95% CI 0.93 to 2.21). Colonic Transit Time (CTT) scores were significantly higher when deep needling was combined with sparse wave (MD −14.36, 95% CI −18.31 to −10.41) or high-frequency dilatational wave (MD −11.53, 95% CI −19.25 to −3.81). The time of first defecation after treatment (TFD) of the shallow needling therapy was significantly longer than that of the lactulose (MD 13.67, 95% CI 5.66 to 21.67). The CCS 6 months after treatment (CCS6m) for deep needling was significantly lower than that for lactulose (MD −4.90, 95% CI −5.97 to −3.84). Moreover, the FSD 6 months after treatment (FSD6m) for shallow needling was significantly higher than that for lactulose (MD 0.49, 95% CI 0.02 to 0.97). The adverse event (AE) rate for lactulose was significantly higher than that achieved with the needling treatments, and this held true for both deep needling therapy (RR 0.41, 95% CI 0.23 to 0.72) and shallow needling therapy (RR 0.33, 95% CI 0.15 to 0.77).

**Conclusions:**

The meta-analysis demonstrates that acupuncture at ST25 appears to be more effective than lactulose in the treatment of functional constipation. This was found to be especially true for deep needling with high-frequency dilatational wave, which had a greater impact on improving CCS, FSD, CTT, and CCS6m. Additionally, acupuncture at ST25 was shown to be safer than conventional treatment, with the rate of AE being significantly lower for both deep needling and shallow needling. The trial is registered with https://www.crd.york.ac.uk/prospero/(CRD42019141017)).

## 1. Introduction

Functional constipation (FC) is a type of general functional gastrointestinal disease. It is characterized by persistent low-frequency defecation, usually accompanied by a tensioning and unpleasant sensation, but without any organic abnormality of the lower abdomen [1, 2]. FC is a common public health issue, with prevalence of about 15.5% in North America [3], 14% in Asia [4], 19.2% in Europe [5], and 19.7% in Oceania [5]. FC is not lethal, but it seriously impacts quality of life and carries a heavy financial burden [6–8]. Common methods of FC management include lifestyle modification and pharmaceuticals. Lifestyle modification, such as physical activities and dietary fiber intake, is recommended to promote bowel movement. However, the efficacy of lifestyle modifications still remains uncertain, and the recommendation strength is weak [9, 10]. Pharmaceuticals which are prescribed to FC patients conventionally, including laxatives, spasmolytics, and gastrointestinal prokinetic agents, have demonstrated efficacy in patients [10]. However, the side effects of the majority of pharmaceuticals, such as dehydration, esophageal obstruction, electrolyte disturbances, and bowel cramps [10], cannot be neglected. Laxatives such as lactulose are often the first-line of defense for FC. Nevertheless, adverse reactions such as flatulence, abdominal pain, nausea, and vomiting are common [11]. Accordingly, seeking a viable alternative cure with less side effects for FC is a priority of both physicians and patients.

Acupuncture is a popular therapy which is often regarded as a complementary and alternative treatment originating from traditional Chinese medicine. It has been used to cure constipation for thousands of years. The efficacy of acupuncture for FC has recently been reported by many high-quality randomized controlled trials (RCTs) [12–16]. Acupuncture can be roughly divided into deep and shallow needling; deep needling, which enters through the peritoneum, can cause severe pain in patients, while shallow needling is not generally painful. Acupuncture is usually combined with a pulse electrotherapy apparatus to strengthen the stimulation of acupoints. Dilatational waves, which are the alternating waveform of sparse waves and dense waves of pulse electric output, have a stronger stimulating effect than sparse waves. However, despite the number of studies, there is still a lack of systematic evaluation of acupuncture depth and waveform in the literature. Tianshu (ST25) [17], a conventional and representative acupoint for constipation [18], has been gaining the growing attention of scholars. Clinical effects are being acknowledged by an increasing number of scholars [19, 20]. The principal mechanism of acupuncture therapy for FC has also become a major focus in acupuncture research [21, 22]. Several studies have demonstrated that acupuncture's mode of treatment for FC is through modulating peripheral gastrointestinal hormones [23], improving gastrointestinal motility [24], and maintaining the balance of excitatory and inhibitory neurons in the enteric nervous system [25].

The aim of this quantitative research study was to collect evidence about the safety and efficacy of acupuncture at ST25 for FC to facilitate the clinical application of this treatment.

## 2. Methods

This study was registered with https://www.crd.york.ac.uk/prospero/(CRD42019141017). It was performed according to the Cochrane Handbook for Systematic Reviews of Interventions [26] and is in accordance with the Preferred Reporting Items for Systematic Reviews and Meta-Analyses (PRISMA) guidelines (Table SM1 in Supplementary Material).

### 2.1. Literature Search

The search terms “functional constipation,” “Dyschezia,” and “Colonic Inertia,” combined with “Tianshu” and “ST25,” were used by two reviewers (PL and YL) in the following databases to search for relevant randomized controlled trials published up to the date of May 5, 2020: PubMed, Embase, the Cochrane Central Register of Controlled Trials (CENTRAL), the China Network Knowledge Infrastructure (CNKI), the Wanfang Data Knowledge Service Platform (Wanfang), the China Biology Medicine disc (CBM), and the China Science and Technology Journal Database (CQVIP). There were no linguistic or geographic restrictions imposed. The detailed search strategy is listed in Table SM2 in Supplementary Material.

### 2.2. Study Selection

The following inclusion criteria were used to select studies: (i) randomized controlled trials (RCTs), regardless of the use of blinding; (ii) studies including patients with functional constipation diagnosed according to Roman criteria [27], including strenuous defecation, hard defecation, incomplete defecation, obstruction of defecation, defecation in need of manual assistance, and reduction of defecation times (regardless of age, sex, and ethnicity); (iii) studies where the experimental group received acupuncture therapy alone while the control group received any conventional therapy except acupuncture, or where the experimental group received acupuncture therapy in combination with conventional therapies while the control group received the same conventional therapy; (iv) studies where Tianshu (ST25) is used as the exclusive acupuncture method; and (v) studies containing data that was reported regarding therapeutic efficacy and safety.

The exclusion criteria were as follows: (i) articles without data on efficacy or safety; (ii) articles of theoretical explorations, case reports, reviews, and animal studies; and (iii) articles with the full text being unavailable.

### 2.3. Data Extraction

Four investigators (PL, YL, QW, and SS) independently chose relevant studies after reading the titles and abstracts. Then, the full texts of the selected studies were further assessed. An additional two researchers (KC and DY) created the self-designed data extraction form, which included general information (i.e., the first author, year, location, study design), participant characteristics (i.e., disease duration, average age, sample size), interventions, course of treatments, main outcomes, adverse events, and recurrence rates. Any disagreements were settled by discussion among the researchers.

### 2.4. Methodological Quality Assessment

Four reviewers (PL, YL, QW, and KC) independently assessed the risk of bias in the included studies by applying the Cochrane risk-of-bias tool and Jadad Scale [28]. The Cochrane risk-of-bias tool evaluated the studies using the following parameters [26]: random sequence generation, allocation concealment, blinding of participants and personnel, blinding of the outcome assessment, incomplete outcome data, selective reporting, and other biases. The results were subsequently classified into low risk, high risk, or unclear risk. Additionally, each study was evaluated by the parameters of the Jadad Scale, which includes the following parameters: randomization, double blinding, withdrawals, and dropouts. The Jadad Scale scores range from zero to five points. Studies with a score less than three were considered low-quality trials, whereas those with a score of greater than or equal to three were considered high-quality trials. The final results were cross-checked by two investigators (SS and CF). Any disagreement was settled by discussion between them.

### 2.5. Data Analyses

Review Manager (RevMan software, version 5.3, Cochrane Collaboration [29]) was used to identify differences in outcome between the experimental (acupuncture) and control groups. Risk ratios (RRs) were calculated for dichotomous data, with a 95% confidence interval (CI). For continuous data, mean differences (MDs) or standard mean differences (SMDs) were calculated, with a 95% CI. The degree of heterogeneity between studies was determined using the *I*^2^ statistic. A fixed model was applied when there was no significant heterogeneity (*I*^2^ < 50%); otherwise, a random effects model was considered suitable. A *P* value of <0.05 was considered statistically significant.

### 2.6. Outcomes

The primary outcome measured was the Cleveland Constipation Score (CCS) [30] which can comprehensively reflect the constipation condition of the patient and the weekly frequency of spontaneous defecation (FSD) after treatment. First, CCS was divided into the following eight categories [30]: frequency of bowel movements, painful evacuation, incomplete evacuation, abdominal pain, length of time per attempt, assistance for defecation, unsuccessful attempts for evacuation per 24 hours, and duration of constipation. A scoring range of 0 to 4 (except for “assistance for defecation,” which used a range of 0 to 2) was derived. Subsequently, the global score was obtained by adding each individual score, and this cumulative score was used to judge the status of constipation comprehensively. Meanwhile, FSD was calculated as the frequency of functional defecation.

Secondary outcomes included the Colonic Transit Time (CTT), the time of first defecation after treatment (TFD), the CCS 6 months after treatment (CCS6m), the FSD 6 months after treatment (FSD6m), the recurrence rate (RER), and the number of adverse events (AE).

## 3. Results

### 3.1. Selection and Characteristics of Studies

**Figure 1:**
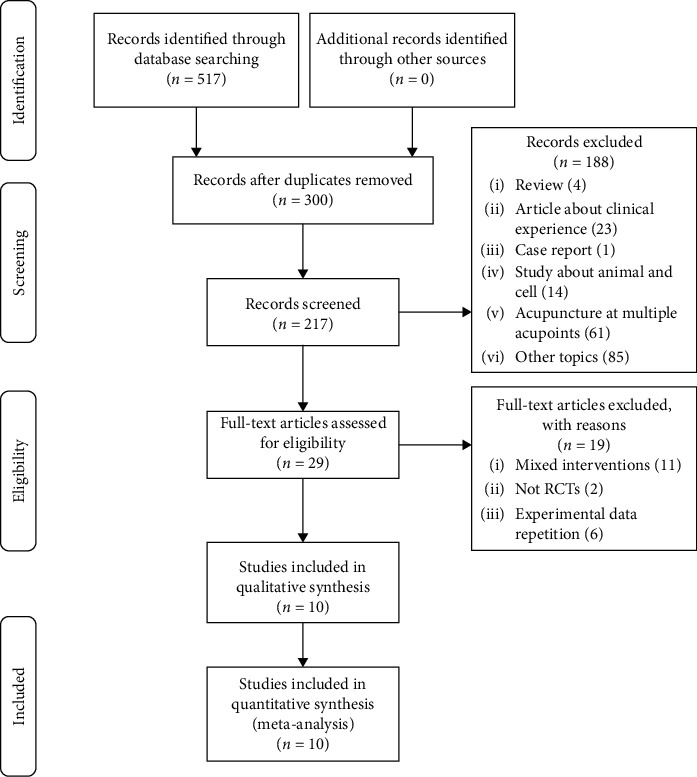
Study selection process for a meta-analysis on the safety and efficacy of acupuncture at Tianshu (ST25) for functional constipation.

Of the 517 initially retrieved studies, 300 were excluded after abstract and full text reviews on the basis that they were duplicate articles, theoretical explorations, case reports, animal trials, fundamental trials, conferences, or reviews. We eliminated two non-RCTs, 11 mixed interventions, and six studies with overlaps in data. Ultimately, 10 RCTs [31–40] met the inclusion criteria and were included in our systematic review, three of which were unpublished master's theses [32, 34, 36] (Figure 1). All included studies were performed in China between 2006 and 2018.

The characteristics of the included trials are listed in Table 1. A total of 1568 patients participated in the studies. Of these, 1108 and 460 patients belonged to the experimental and control groups, respectively. All patients met the diagnosis of functional constipation according to Rome III criteria [27]. Eight of the studies [32–39] included two experimental groups (deep needling and shallow needling therapy) and one control group, in which seven studies [33–39] used electroacupuncture stimulation to attach the needle with low-frequency dilatational wave. One study [32] chose the pattern of high-frequency dilatational wave. The remaining two studies [31, 40] included an experimental group (deep needling) and a control group, which employed the sparse wave pattern. Control groups for all studies were treated with lactulose. Only one study [32] reported recurrences and calculated RERs. Nine studies [31, 32, 34–40] reported AEs. All studies reported CCS, four studies [33, 35, 37, 39] reported FSD, five studies [31, 32, 34, 35, 40] reported CTT, two studies [32, 34] reported TFD, three studies [31, 36, 40] reported CCS6m, and two studies [32, 37] reported FSD6m.

### 3.2. Risk of Bias

**Figure 2:**
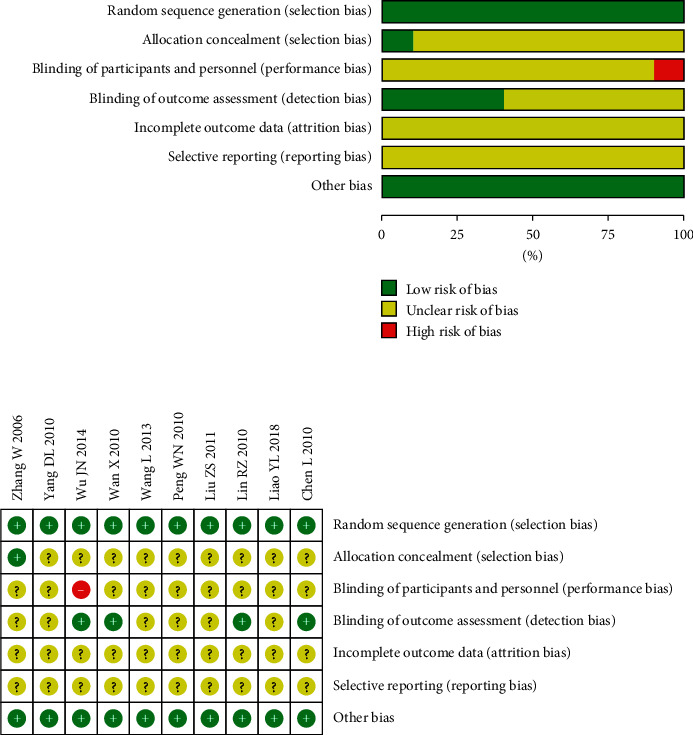
Risk of bias in the studies included in a meta-analysis on the safety and efficacy of acupuncture at Tianshu (ST25) for functional constipation. (a) Risk-of-bias graph. (b) Risk-of-bias summary.

Most of the included trials were assessed to be of generally high methodological quality. The risks of bias in all 10 studies are shown in Figure 2. Each study was described as randomized by the authors, and all of them stated methods of random sequences generation, seven of which [32, 35, 37, 39] were based on central randomization, and the remaining three [31, 36, 40] of which were based on tables of random numbers that were generated by a computer. Only one trial [31] reported allocation concealment which was assigned through an opaque envelope but did not mention blinding method of the outcome assessment. Another trial [39] stated that blinding was not performed on patients or acupuncturists unequivocally, so we judged that the blinding of participants and personnel was high risk for introducing bias. Four trials [32, 34, 36, 39] had a complete and detailed description of final assessment, so we regarded this as having a low risk of bias. Among the remaining trials, allocation concealment, blinding of participants and personnel, and blinding of outcome assessment were not mentioned. Since the protocols of all 10 included trials were not accessible, incomplete outcome data and selective reporting were generally unclear. Jadad scores of 10 trials ranged from two to five. However, interrater agreement on methodological quality was acceptable (Table 2). The characteristics of participants in the different treatment groups of each study were comparable in terms of baseline (gender, age, severity of disease, etc.).

### 3.3. Primary Outcomes

#### 3.3.1. CCS

**Figure 3:**
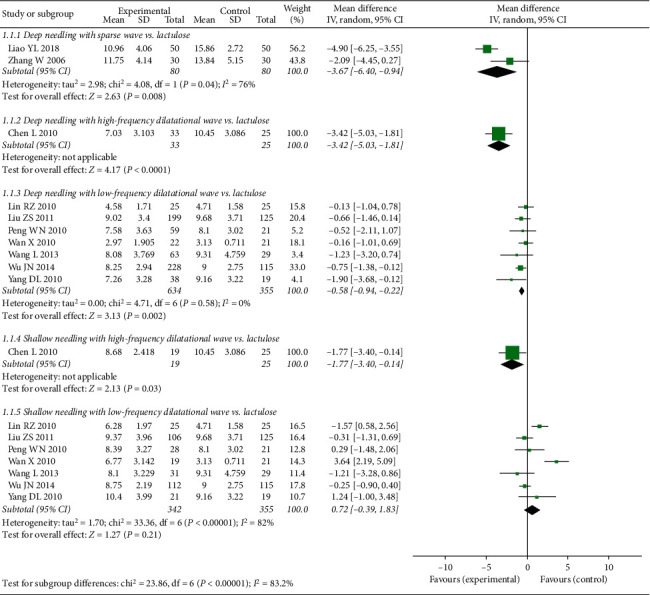
Forest plot comparing Cleveland Constipation Score (CCS) between needling group and control group for patients with functional constipation.

Subgroup analysis demonstrated that CCS for deep needling therapy was significantly lower than that for lactulose (deep needling with low-frequency dilatational wave vs. lactulose: MD −0.58, 95% CI −0.94 to −0.22, *P*=0.002; deep needling with sparse wave vs. lactulose: MD −3.67, 95% CI −6.40 to −0.94, *P*=0.008; deep needling with high-frequency dilatational vs. lactulose: MD −3.42, 95% CI −5.03 to −1.81, *P* < 0.0001). Moreover, CCS for shallow needling with high-frequency dilatational wave was lower than that for lactulose (shallow needling with high-frequency dilatational wave vs. lactulose: MD −1.77, 95% CI −3.40 to −0.14, *P*=0.03). However, the CCS for shallow needling with low-frequency dilatational wave was comparable with that of lactulose (shallow needling with low-frequency dilatational wave vs. lactulose: MD 0.72, 95% CI −0.39 to 1.83, *P*=0.21; see Figure 3).

#### 3.3.2. FSD

**Figure 4:**
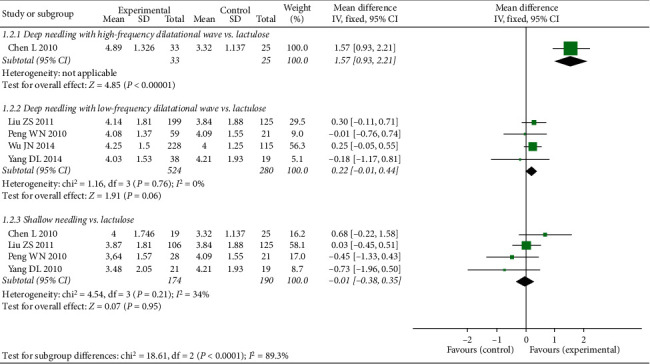
Forest plot comparing the weekly frequency of spontaneous defecation (FSD) between needling group and control group for patients with functional constipation.

Subgroup analysis demonstrated that FSD for deep needling with high-frequency dilatational wave was significantly higher than that for lactulose (deep needling with high-frequency dilatational wave vs. lactulose: MD 1.57, 95% CI 0.93 to 2.21, *P* < 0.00001). However, the FSD for deep needling with low-frequency dilatational wave and shallow needling therapy was comparable with that for lactulose (deep needling with low-frequency dilatational wave vs. lactulose: MD 0.22, 95% CI −0.01 to 0.44, *P*=0.06; shallow needling vs. lactulose: MD −0.01, 95% CI −0.38 to 0.35, *P*=0.95; see Figure 4).

### 3.4. Secondary Outcomes

#### 3.4.1. CTT

**Figure 5:**
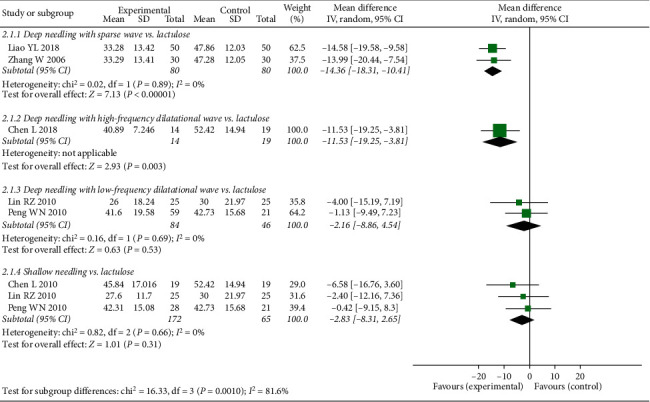
Forest plot comparing Colonic Transit Time (CTT) between needling group and control group for patients with functional constipation.

We used CTT to assess the direct curative effects of the therapies. The trend for CTT was similar to that of FSD. We found that CTT was significantly higher when deep needling was combined with sparse wave or high-frequency dilatational wave (deep needling with sparse wave vs. lactulose: MD −14.36, 95% CI −18.31 to −10.41, *P* < 0.00001; deep needling with high-frequency dilatational wave vs. lactulose: MD −11.53, 95% CI −19.25 to −3.81, *P*=0.003). However, the CTTs for deep needling with low-frequency dilatational wave and shallow needling therapy were comparable with that for lactulose (deep needling with low-frequency dilatational wave vs. lactulose: MD −2.16, 95% CI −8.86 to 4.54, *P*=0.53; shallow needling therapy vs. lactulose: MD −2.83, 95% CI −8.31 to 2.65, *P*=0.31; see Figure 5).

#### 3.4.2. TFD

**Figure 6:**
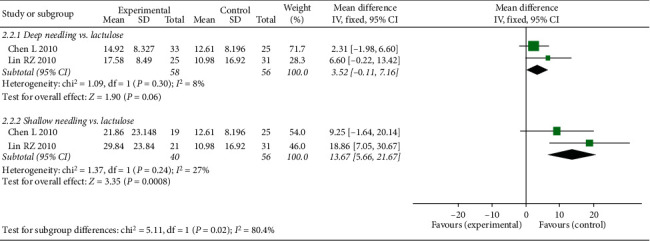
Forest plot comparing the time of first defecation after treatment (TFD) between needling group and control group for patients with functional constipation.

TFD was used to evaluate the effect time in the different treatment groups. The TFD for deep needling was comparable with that for lactulose (deep needling vs. lactulose: MD 3.52, 95% CI −0.11 to 7.16, *P*=0.06). However, the TFD of the shallow needling therapy was significantly longer than that of the lactulose (shallow needling therapy vs. lactulose: MD 13.67, 95% CI 5.66 to 21.67, *P*=0.0008; see Figure 6).

#### 3.4.3. CCS6m

**Figure 7:**
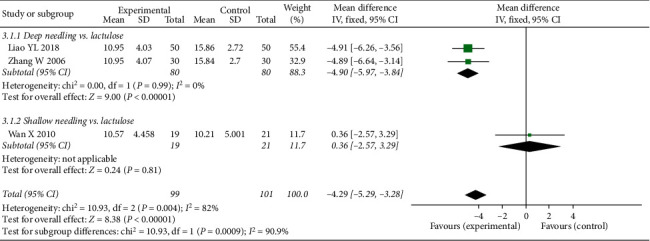
Forest plot comparing the Cleveland Constipation Score of 6 months after treatment (CCS6m) between needling group and control group for patients with functional constipation.

CCS6m was used to evaluate the long-term effects in the different treatment groups. The CCS6m for deep needling was significantly lower than that for lactulose (deep needling vs. lactulose: MD −4.90, 95% CI −5.97 to −3.84, *P* < 0.00001). Nonetheless, the CCS6m of shallow needling was comparable with that of lactulose (shallow needling therapy vs. lactulose: MD 0.36, 95% CI −2.57 to 3.29, *P*=0.81; see Figure 7).

#### 3.4.4. FSD6m

**Figure 8:**
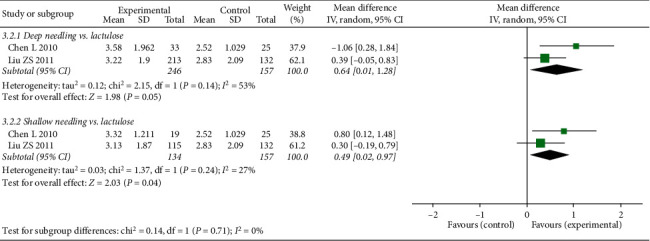
Forest plot comparing the weekly frequency of spontaneous defecation of 6 months after treatment (FSD6m) between needling group and control group for patients with functional constipation.

Subgroup analysis demonstrated that FSD6m for deep needling was comparable with lactulose treatment (deep needling vs. lactulose: MD 0.64, 95% CI 0.01 to 1.28, *P*=0.05). However, the FSD6m for shallow needling was significantly higher than that for lactulose (shallow needling vs. lactulose: MD 0.49, 95% CI 0.02 to 0.97, *P*=0.04; see Figure 8).

#### 3.4.5. RER

**Figure 9:**
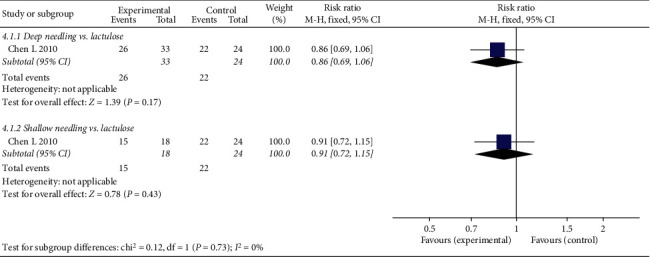
Forest plot comparing recurrence rates between needling group and control group for patients with functional constipation.

Only one trial reported RER.  Figure 9 shows that there was no significant difference in RER between the experimental and control groups (deep needling vs. lactulose: RR 0.86, 95% CI 0.69 to 1.06, *P*=0.17; shallow needling vs. lactulose: RR 0.91, 95% CI 0.72 to 1.15, *P*=0.43; see Figure 9).

#### 3.4.6. AE

**Figure 10:**
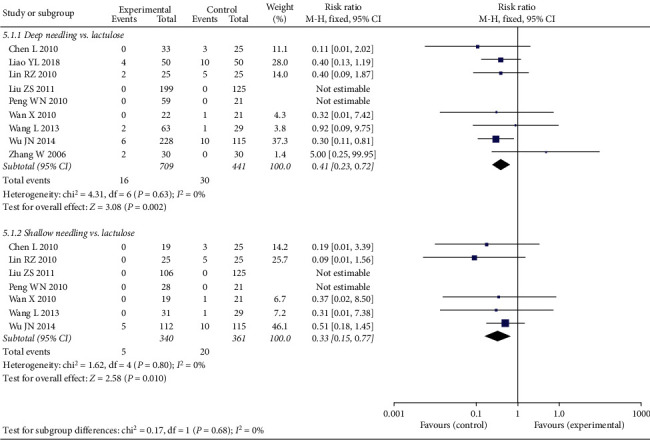
Forest plot comparing adverse events between needling group and control group for patients with functional constipation.

The AE rate for lactulose was significantly higher compared to both types of needling treatments (deep needling vs. lactulose: RR 0.41, 95% CI 0.23 to 0.72, *P*=0.002; shallow needling vs. lactulose: RR 0.33, 95% CI 0.15 to 0.77, *P*=0.010; see Figure 10).

## 4. Discussion

We were able to demonstrate to a limited extent the safety and efficacy of acupuncture at ST25 for function al constipation, even though the quality of the 10 RCTs which were included in the present study was not ideal. Regardless of the waveform of electroacupuncture or the depth of acupuncture, the study was able to comprehensively demonstrate that the CCS for deep needling was significantly lower than that for lactulose (control) group, and thus the condition of the patient was improved. Moreover, the FSD for deep needling with high-frequency dilatational wave was remarkably higher than that for lactulose; however, evidence for this is inadequate because this direct comparison was made in only one study [32]. It is well known that lactulose can increase intestinal osmotic pressure and promote defecation effectively for functional constipation in modern medicine [41]. Nevertheless, the chronic use of lactulose can cause flatus and bacterial adaptation [41–43]. In addition, our study showed that the FSD for lactulose was not significantly different from the frequency for deep needling with low-frequency dilatational wave, shallow needling with high-frequency dilatational wave, or shallow needling with low-frequency dilatational wave.

However, of note is that the FSD significantly increased for deep needling therapy with high-frequency dilatational wave. CTT scores showed a similar trend with a significant improvement, and this was only when deep needling was combined with sparse wave and high-frequency dilatational wave. These findings suggest that deep needling at ST25 may promote defecation by reducing CTT. Modern studies have shown that the nerve segments of the colon are T10 to L3 and the sacral plexuses, and the nerve segment of ST25 is T10 [44]. Additionally, it has been shown that the afferent impulses of acupuncture at ST25 can be projected to the colon [44]. Subsequently, the afferent impulse of acupuncture at ST25 is transmitted through two pathways of the somatic nerve and vascular wall nerve plexus, through the spinal cord, to all the levels of centers of cerebral cortex and connects with the viscera, which then transmit it through the autonomic nervous system and bodily fluid to regulate gastrointestinal function [45]. This significantly increases the amplitude and frequency of the distal colon [46], strengthens the tension of the colon, makes the contraction more powerful, and is thus beneficial to defecation [47].

The long-term effect of defecation improvement is as important as the short-term effect for FC patients. The pathogenesis of FC is mainly related to colonic motility disorder, perianal sphincter dysfunction, and abnormal pathological changes of intestinal hormones. Gastrointestinal hormones mainly include excitatory transmitters such as acetylcholine (ACh) and substance P, as well as inhibitory transmitters such as nitric oxide (NO), vasoactive intestinal peptide (VIP), and adenosine triphosphate (ATP) [36]. Recently, it has been found that interstitial Cajal cell (ICC) is a pacemaker of slow waves in the intestinal tract, which is involved in the process of information transmission in the intestinal noncholinergic nervous system (NANC) and has the potential role of controlling gastrointestinal motility. It is also related to the pathogenesis of human gastrointestinal motor disease [48, 49]. There was irregular distribution of ICC in the whole colon tissue in patients with functional constipation, and the volume of ICC was significantly reduced [50]. Acupuncture at ST25 can cause severe pain, which triggers a large number of pain receptors, and these excite unmyelinated III (AS) and IV (C) afferent fibers and central nervous system fibers to activate the brainstem. It produces bidirectional regulation of neurophysiology [51] and can regulate increased secretion of neurotransmitters such as acetylcholine (ACh), substance P, and nitric oxide (NO). At the same time, acupuncture stimulation may promote the repair of colonic neuromuscular tissue, especially in the repair of ICC [36]. These comprehensive factors may be the physiological basis of acupuncture at ST25 in the treatment of functional constipation, which is beneficial to patients not only in the short term, but also in the long term.

However, no matter which treatment is used, recurrence cannot be ignored, which is comparable in the control group and the experimental group. This suggests that acupuncture may not increase RER. Although the AE of acupuncture at ST25 was lower than that of lactulose, the AE of pain could not be ignored. The included studies [31, 34, 38–40] showed that the side effects of the needling group could disappear within a short limited period of time after treatment of symptoms or without any special treatment. Future research should explore a combination of multiple acupoints, combined with acupuncture anesthesia technology, to develop a treatment scheme with better curative effect and less pain.

There are some limitations to this study. Firstly, the sample size is not large enough to draw reliable conclusions, and the quality of the included trials was not overwhelmingly high. Of the 10 trials, only one trial [31] reported an allocation concealment method. Additionally, none of the studies involved blinding of the researchers and participants. Secondly, all the studies were conducted in China, and the results may vary according to ethnicity or diet. Thirdly, the number of events in some subgroups that the results were based on was exceedingly small, which may affect the interpretations.

## 5. Conclusions

In conclusion, acupuncture at ST25 may be more effective than lactulose for functional constipation. In particular, deep needling with high-frequency dilatational wave had a substantial impact on improving CCS, FSD, CTT, and CCS6m. In terms of safety, the rate of AE for acupuncture with both deep and shallow needling was significantly lower than that for lactulose. However, a larger number of high-quality RCTs with a lower risk of bias and adequate sample sizes are required to confirm the results of this quantitative research study.

## Figures and Tables

**Table 1:** Characteristics of the included trials in a quantitative study of the efficacy and safety of acupuncture ST25 for functional constipation.

Study	Location	Baseline data comparable	Sample size		Average age (years)		Course of treatment (weeks)	Adverse events		Disease duration(years)		Interventions		EA	Main outcomes
			E	C	E	C		E	C	E	C	E	C		
Zhang, 2006 [31]	China	Yes	30	30	65	67	2	Pain (2)	0	13.4 (8.62)	11.00 (5.59)	Deep needling	Lactulose	Sparse wave	CCS, CTT, CCS6m, AE

Chen, 2010 [32]	China	Yes	33/19	25	43.56 (18.597)/44.14 (16.571)	47.61 (18.901)	4	0	Diarrhea (3)	10.05 (8.95)/9.24 (9.41)	12.56 (11.46)	Deep needling/shallow needling	Lactulose	High-frequency dilatational wave	CCS, CTT, TFD, FSD6m, AE, RER

Yang and Liu, 2010 [33]	China	Yes	38/21	19	53.1/57	50.1	4	NR	NR	12.67/12.71	10.62	Deep needling/shallow needling	Lactulose	Low-frequency dilatational wave	CCS, FSD

Lin, 2010 [34]	China	Yes	25/25	25	20.68 (2.27)/21.44 (2.10)	21.24 (1.45)	4	Pain (2)	Abdominal distension (5)	3.36 (2.20)/4.32 (1.77)	4.44 (2.10)	Deep needling/shallow needling	Lactulose	Low-frequency dilatational wave	CCS, CTT, TFD, AE

Peng et al., 2010 [35]	China	Yes	59/28	21	53.10/50.14	59.24	4	0	0	10.93/9.65	7.78	Deep needling/shallow needling	Lactulose	Low-frequency dilatational wave	CCS, FSD, CTT, AE

Wan, 2010 [36]	China	Yes	22/19	21	23.74 (7.02)/22.03 (5.05)	25.18 (8.78)	4	0	Diarrhea (1)	NR	NR	Deep needling/shallow needling	Lactulose	Low-frequency dilatational wave	CCS, CCS6m, AE

Liu et al., 2011 [37]	China	Yes	199/106	125	NR	NR	4	0	0	NR	NR	Deep needling/shallow needling	Lactulose	Low-frequency dilatational wave	CCS, FSD, FSD6m, AE

Wang, 2013 [38]	China	Yes	63/31	29	53.29 (13.45)/58.85 (12.04)	51.61 (16.61)	4	Pain (2)	Abdominal pain, diarrhea, and dizziness (1)	10.4 (10.7)/8.15 (10.25)	9.83 (8.82)	Deep needling/shallow needling	Lactulose	Low-frequency dilatational wave	CCS, AE

Wu et al., 2014 [39]	China	Yes	228/112	115	45.88 (16.85)/46.25 (16.81)	44.12 (17.48)	4	Pain, fatigue, or subcutaneous hemorrhage (11)	Diarrhea or abdominal discomfort (10)	9.24 (8.32)/9.10 (8.38)	9.25 (9.18)	Deep needling/shallow needling	Lactulose	Low-frequency dilatational wave	CCS, FSD, AE

Liao, 2018 [40]	China	Yes	50	50	64.98 (3.51)	65.36 (3.45)	2	Headache (1), dizziness (1), gastrointestinal discomfort (2)	Headache (3), dizziness (4), gastrointestinal discomfort (3)	NR	NR	Deep needling	Lactulose	Sparse wave	CCS, CTT, CCS6m, AE

E: experimental group (deep needling therapy or shallow needling therapy), C: control group (lactulose therapy), NR: no report, RCT: randomized controlled trial, EA: electroacupuncture, CCS: Cleveland Constipation Score, FSD: weekly frequency of spontaneous defecation, CTT: Colonic Transit Time, TFD: time of first defecation after treatment, CCS6m: Cleveland Constipation Score of 6 months after treatment, FSD6m: weekly frequency of spontaneous defecation of 6 months after treatment, RER: recurrence rate, AE: adverse event.

**Table 2:** The Jadad score for assessing risk of bias.

Study (first author, year)	Random sequence generation	Double blinding	Withdrawals and dropouts	Jadad score
Zhang, 2006 [31]	2	0	0	2
Chen, 2010 [32]	2	1	1	4
Yang and Liu, 2010 [33]	2	0	1	3
Lin, 2010 [34]	2	1	0	3
Peng et al., 2010 [35]	2	0	1	3
Wan, 2010 [36]	2	1	1	4
Liu et al., 2011 [37]	2	0	0	2
Wang, 2013 [38]	2	0	1	3
Wu et al., 2014 [39]	2	2	1	5
Liao, 2018 [40]	2	0	0	2

## Data Availability

The data used to support the findings of this study are included within the article and the supplementary information files.

## Abbreviations

FC: Functional constipation
RR: Risk ratio
MD: Mean difference
SMD: Standard mean difference
CI: Confidence interval
CCS: Cleveland Constipation Score
FSD: Weekly frequency of spontaneous defecation
CTT: Colonic Transit Time
TFD: The time of first defecation after treatment
CCS6m: Cleveland Constipation Score of 6 months after treatment
FSD6m: Weekly frequency of spontaneous defecation of 6 months after treatment
RER: Recurrence rate
AE: Adverse event
RCT: Randomized controlled trial
CENTRAL: Cochrane Central Register of Controlled Trials
Embase: Excerpta Medica database
CNKI: China Network Knowledge Infrastructure
Wanfang: Wanfang Data Knowledge Service Platform
CBM: China Biology Medicine disc
CQVIP: China Science and Technology Journal Database
ICC: Interstitial Cajal cell
E: Experimental group
C: Control group
EA: Electroacupuncture
NR: No report.

## Supplementary Materials

Supplementary Materials. Table SM1: PRISMA checklist. Table SM2: search strategy.
